# A new performance-based measure of personality functioning impairment: development and preliminary evaluation of reliability and validity

**DOI:** 10.1007/s44192-024-00059-4

**Published:** 2024-02-22

**Authors:** Adam P. Natoli, Chloe M. Rodriguez

**Affiliations:** Department of Psychology and Philosophy, Sam Houston State University, Huntsville, TX USA

**Keywords:** Personality functioning impairment, AMPD, Implicit association test, Psychometrics

## Abstract

Personality functioning impairment is at the center of many dimensional models of personality. Available measures of personality functioning impairment are limited to self-report, clinician-/informant-rated, and interview methods. Although researchers have begun investigating established performance-based instruments’ potential for assessing personality functioning impairment, administration and scoring of these instruments is complex and the latent variables they measure diverge from personality functioning impairment as described in the ICD-11 and the Alternative Model for Personality Disorders (AMPD) of the DSM. We address this absence by developing and psychometrically evaluating the Level of Personality Functioning Scale-Questionnaire-based Implicit Association Test (LPFS-qIAT). The LPFS-qIAT’s psychometric properties were evaluated across four studies, producing initial evidence supporting the new instrument’s reliability as well as its convergent, discriminant, and criterion-related validity. As the first performance-based measure of personality functioning impairment that aligns with the AMPD and, to a degree the ICD-11, that is easily administered, scored, and interpreted, the LPFS-qIAT shows potential to become a valuable tool in both research and clinical practice.

**Supplementary Information:**

The online version contains supplementary material available at 10.1007/s44192-024-00059-4.

The global prevalence rate of personality disorders is fairly high (ranging from 7.8% to 12.16%), with personality disorders showing links to high health service utilization [1], economic burden [2, 3], medical morbidity and mortality [4], double the odds of poor treatment outcome [5], multiple public health issues [6, 7], and societal costs [8]. Although some mental health professionals and researchers still use a categorical model of personality pathology, recent shifts in diagnostic systems and the growing body of evidence supporting the scientific [9] and practical [10] superiority of dimensional models will inevitably lead to the adoption of the latter. Most dimensional models conceptualize personality in terms of functioning and style and consider personality functioning impairment the core of personality pathology. Consequently, there will be an increased need to assess personality functioning impairment accurately and easily as the field continues to shift toward the use of dimensional models. Numerous measures of personality functioning impairment have been developed (see [9, 11]). Available instruments overwhelmingly use self-report, clinician-/informant-rating, or interview methods of measurement. Some researchers have begun investigating different performance-based instruments’ potential for assessing personality functioning impairment [12]; however, the administration and scoring of these instruments is habitually complex, time intensive, and the latent variable(s) measured diverge from personality functioning impairment as described in the chapter on personality disorders and related traits in the 11th edition of the *International Classification of Diseases* (ICD-11) [13] and in the D*iagnostic and Statistical Manual of Mental Disorders’* (DSM) Alternative Model for Personality Disorders (AMPD) [14, 15]. To address the prominent absence of an easily administered and scored performance-based measure of personality functioning impairment that aligns with these diagnostic systems, we developed a novel instrument that meets these criteria and directly parallels the AMPD’s Level of Personality Functioning Scale (LPFS) [14]. The new instrument’s internal and retest reliability and several aspects of validity were then evaluated through four separate studies.

## Background

Over the last decade, classification systems of personality disorders have refined their diagnostic frameworks by introducing dimensional models intended to address the many limitations of longstanding categorical models [16–19]. Perhaps the clearest examples of this shift are the ICD-11’s [13] removal of all personality disorder categories and the introduction of the AMPD in DSM-5 [14]. Consideration of severity—level of personality functioning impairment—is essential to any dimensional model of personality [20, 21] and research has revealed generalized severity of functioning to be one of the most robust and important single predictors of personality pathology [22]. Accordingly, the dimensional models of the ICD-11 and DSM both emphasize the importance of assessing personality functioning impairment. Yet, measures of personality functioning are informative in matters beyond diagnosis; personality functioning spans a continuum from healthy, adaptive functioning to severe impairment and its measurement is relevant whenever one wishes to understand an individual’s psychology. Fortunately, numerous instruments have been developed for measuring personality functioning.

### Measures of personality functioning impairment

Most measures of personality functioning impairment make use of self-report methods (see [9, 11, 23, 24] for general and psychometric reviews). The collection of self-report measures include the Levels of Personality Functioning-Brief Form 2.0 (LPFA-BF 2.0) [25], Levels of Personality Functioning Scale-Self-Report (LPFS-SR) [26], DSM-5 Levels of Personality Functioning Questionnaire (DLOPFQ) [27] and a corresponding short-form (DLOPFQ-SF) [28], Self and Interpersonal Functioning Scale (SIFS) [29], Levels of Personality Functioning Scale-Self-Report of Criterion A (LPFS-SRA) [30], the Personality Disorder Severity ICD-11 scale (PDS-ICD-11) [31], and Levels of Personality Functioning Questionnaire for Adolescents from 12 to 18 years (LoPF-Q 12–18) [32]. These instruments range in length (from 12 to 132 items), in number of scales, and in the amount of psychometric evidence supporting their use. Largely, psychometric investigations of self-report measures of personality functioning impairment have supported the validity, internal consistency, factor structure, and measurement invariance of these instruments in several diverse populations and for an assortment of external criteria. To the authors’ knowledge, the only^1^ study to date to investigate the retest reliability of a self-report measure of personality functioning impairment demonstrated good to excellent reliability of the LPFS-SR over approximately 2 weeks (*r*s = 0.81–0.91) [33].

Complementing self-report tests of personality functioning impairment are a small group of (semi-)structured interviews and informant- and clinician-rated measures (see [9, 11] for reviews]. Many of the above self-report instruments were based on the original LPFS [14], which was introduced in the AMPD as a clinician-rated scale designed to help assess personality disorder severity in a dimensional manner [34]. The LPFS has proven to be a valid measure of personality functioning impairment [11, 34] with interrater reliability ranging from adequate to good (ICC = 0.7–0.9) [24]. Prevailing interview-based measures of personality functioning impairment include the Semi-Structured Interview for Personality Functioning DSM-5 (STiP-5.1) [35], the Structured Clinical Interview for the Level of Personality Functioning Scale (SCID-AMPD Module 1) [36], the Clinical Assessment of the Level of Personality Functioning Scale (CALF) [37], and the PDS-ICD-11 Clinician-Rating Form [38]. Designed as (semi-)structured interviews, these instruments range in duration, from ~ 30 min to upwards of 45–90 min. Multiple studies have demonstrated their criterion-related and predictive validity, while internal consistency is regularly good and interrater reliability has ranged from 0.59 to 0.96 across the various instruments and their subscales.

As shown by this brief summary, there are myriad self-report, interview, and clinician-/informant-rated measures of personality functioning impairment. There has yet to be a valid performance-based instrument specifically developed to measure personality functioning impairment as defined in the AMPD or ICD-11. Although researchers have demonstrated the potential for using the Rorschach [39, 40] and Thematic Apperception Test [41, 42] to assess personality functioning impairment, reliable and valid administration and scoring of these tests is time-intensive, requires extensive training, and is often incompatible with the type of remote administration required for online studies. Moreover, the constructs (currently) assessed via the Rorschach and Thematic Apperception Test conceptually diverge from personality functioning impairment as operationalized in the AMPD and ICD-11. There is a discernable absence of a performance-based measure of personality functioning impairment that is psychometrically robust, aligns with the AMPD and ICD-11, and can be administered and scored just as quickly, easily, and remotely as self-report tests. The lack of such an instrument is deleterious for both research and clinical practice because overreliance on a single method of measurement can lead to flawed empirical and clinical conclusions based on biased or incomplete representations of personality (see [43–46] for discussions). There is a clear need for a psychometrically sound performance-based measure of personality functioning impairment that aligns with the AMPD and ICD-11 and that can be easily, quickly, and remotely administered and scored.

### Performance-based measurement methodology and the questionnaire-based IAT

Performance-based measures are a broad class of instruments wherein outcomes are derived from an individual’s unrehearsed performance on a (semi-)structured task designed to tap behavior and patterns of responding that reflect—or are influenced by—the latent construct being measured. Information-processing measures are a subtype of the performance-based class. These tests require individuals to complete a reaction-time task by which latent constructs are assessed with the understanding that attributes of the latent construct influence the individual’s task performance in measurable ways via cognitive and other psychological processes largely occurring outside their conscious awareness. The Implicit Association Test (IAT) [46] is one such instrument. The IAT and its variants measure the strengths of implicit—relatively automatic—associations between concepts using a double-categorization reaction-time task (see [46] for further explanation). Intraindividual differences in performance speeds (latencies) between critical test blocks are the basis of IAT scores, with scores representing the strength of automatic associations between the measured concepts in the individual’s mind. With regard to measuring personality functioning impairment, IAT scores would be interpreted as the degree to which the individual associates their concept of themselves with the latent variable of personality dysfunction.

Findings from studies of the IAT’s psychometric properties are fairly variable. Meta-analysis [47] produced an average internal consistency of *α* = 0.80 for the original IAT method, with average alphas of IAT variants ranging from 0.60 to 0.79. Test–retest reliabilities for the IAT averaged *r* = 0.50 across 58 studies, a result Greenwald and Lai [47] partly attributed to reliability reductions caused by operational requirements of certain research situations, such as those seen with online data collection. Regarding construct validity, meta-analytic studies have found aggregate correlations between the IAT and self-reports measuring analogous constructs to range from *r* = 0.116 to *r* = 0.361 and aggregate correlations between IAT measures and external criteria to range from *r* = 0.097 to *r* = 0.274 [48–52]. These effect sizes are comparable to those commonly observed between well-validated performance-based measures (e.g., Rorschach) and scores obtained via self-report or informant-rating scales (correlations typically in the *r* = 0.20–0.30 range) [53–56]. Taken together, psychometric benchmarks for a new performance-based measure of personality functioning impairment based on the IAT methodology can be judiciously set at (a) internal reliability coefficients of approximately *α* = 0.80 or better, (b) test–retest reliability better than *r* = 0.50,^2^ and (c) correlation coefficients representing convergence with validated self-report and informant-rating tests within the *r* = 0.20–0.30 range or stronger, in addition to (d) evidence of discriminant validity.

Although a personality functioning impairment IAT might meet the psychometric benchmarks enumerated above, many of the IAT’s psychometric shortcomings are worsened when it is used to measure self-concepts due to various limitations (e.g., problematic response stimuli, vague and not easily translatable interpretations of scores, and restrictions on the complexity of the constructs that can be measured) [57]. The Questionnaire-based IAT (qIAT) [58, 59] was developed to address these specific barriers. The qIAT procedure is consistent with that of the original IAT but supplements the weaknesses listed above in several ways. Most notably, the qIAT uses items of validated self-report tests as stimuli and creates pairing combinations that parallel self-report procedures (e.g., true/false). Rather than single words or images, qIAT stimuli are statements resembling those found on self-report tests (e.g., “*I often think very negatively about myself*”, “*My emotions are usually well regulated and stable*”). Respondents are instructed to classify these statements as quickly and accurately as possible into relevant target categories (e.g., “*impaired*” versus “*not impaired*”, respectively) while concurrently classifying objective self-statements (e.g., “*I am doing a psychology experiment*”, “*I am playing football on the grass*”) into the categories of “*true*” or “*false*”. Despite unmistakable similarities with self-report measures, scores derived from the qIAT reflect an individual’s automatic association between themselves and the concept being measured whereas self-report scores reflect the degree to which an individual thoughtfully attributes characteristics of a given trait, feeling, thought pattern, motive, behavior, level of functioning, or experience to themselves. That is, self-report scores quantify how the person views and (chooses to) presents themselves whereas qIAT scores would quantify how strongly the latent construct is connected to the individual’s implicit self-conceptualization.

Like self-report tests, the qIAT is particularly well-suited for both research and clinical practice due to its brief administration (~ 5–10 min), simple administration and automatic scoring procedures, direct interpretability of scores (an important feature for clinical utility), and use of validated and standardized stimuli. It is for these reasons that the collection of studies reported herein aimed to develop and psychometrically evaluate a novel, performance-based measure of personality functioning impairment using the qIAT methodology.

### Current study

The overarching goal of the reported series of studies was to develop the Level of Personality Functioning Scale-qIAT (LPFS-qIAT) and conduct an initial psychometric evaluation of this new instrument. Following development of the LPFS-qIAT (see Sect. 8 below for details), four studies were conducted to examine the LPFS-qIAT’s internal and retest reliability and the convergent, discriminant, and criterion-related validity of this new instrument.

## Method

### Participants

All results reported below were obtained using participants recruited through four separate online studies conducted over the course of approximately two years at a medium-sized public university in the Southern United States. Participation was restricted to students who were at least 18 years of age and English-speaking. All participants were required to complete the online studies on a device with a keyboard, which was necessary for administration of the IAT and qIAT measures. Participants were notified at the outset of their participation if they attempted a study on an incompatible device (e.g., touchscreen phone) and were asked to switch to a compatible device, at which point they were permitted to begin the study. No other restrictions were placed upon participation.

Similar procedures were used in each of the four studies: Following informed consent, participants were asked to complete a series of self-report and performance-based measures in a randomized order before completing an extended demographic questionnaire. For those studies including informant ratings and a second participation session (Samples 3 and 4), participants were asked to provide their own email address and the email addresses of two (Sample 3) or three (Sample 4) individuals who know them reasonably well. Informants were automatically emailed a study invitation immediately following a participant’s provision of their email address. Participants were automatically emailed a Time 2 study invitation exactly one (Sample 3) or two (Sample 4) weeks following their survey completion at Time 1. Each study received approval from the Institutional Review Board at the authors’ university and all participants received course credit for participating. The following describes the conventional data processing procedures that were followed for each study and descriptive statistics of each sample are reported in Table 1. Results of group comparisons between those who completed the full study and those who dropped out or were removed due to excessively fast responding on the IAT or qIAT can be found in the Supplementary Material of this paper.

- ***Sample 1***. A total of 1043 participants completed the first study and endorsed taking part in the study seriously and honestly. Three participants did not complete the LPFS-qIAT and 234 participants were removed due to excessively fast responses (see “Data processing” section below), a drop rate within ranges commonly seen for anonymous online data collections [60]. Thus, the final sample included 806 participants.
- ***Sample 2***. A total of 708 students participated in the second study and endorsed taking part in the study seriously and honestly. The final sample included 497 participants after removing 211 participants due to excessively fast responding on the qIAT measure.
- ***Sample 3*.** A total of 287 participants completed Time 1 of the third study and 156 (54%) completed Time 2 and endorsed taking part in the studies seriously and honestly. An additional 78 participants from Time 1 and 41 participants from Time 2 were removed due to excessively fast responding. This resulted in final samples of 209 participants for Time 1 and 115 participants for Time 2. Usable data for both Time 1 and Time 2 was provided by 104 participants.
- ***Sample 4*.** A total of 320 participants completed Time 1 of the fourth study and 78 (24%) completed Time 2, and endorsed taking part in the studies seriously and honestly. Eighty-four participants from Time 1 and 22 participants from Time 2 were removed due to excessively fast responding. This resulted in final samples of 236 participants for Time 1 and 56 participants for Time 2. Forty-eight participants provided usable data for both Time 1 and Time 2.

**Table 1:** Descriptive statistics of all samples

	Sample 1	Sample 2	Sample 3 (time 1)	Sample 3 (time 2)	Sample 4 (time 1)	Sample 4 (time 2)
	(*N* = 806)	(*N* = 497)	(*N* = 209)	(*N* = 104)	(*N* = 236)	(*N* = 48)
Age						
μ (SD)	21.19 (4.83)	21.12 (4.91)	21.82 (6.88)	21.58 (7.23)	20.60 (4.58)	21.17 (5.22)
Range	18–57	18–57	18–56	18–56	18–58	18–44
Sex (%)						
Female	82.98%	85.71%	86.60%	87.13%	86.44%	89.58%
Male	16.52%	13.88%	12.92%	11.88%	12.71%	10.42%
Other/I wish not to respond	0.50%	0.40%	0.48%	0.99%	0.85%	0.00%
Ethnicity (%)						
Hispanic/Latine	31.39%	34.00%	29.19%	29.70%	36.44%	41.67%
Non-Hispanic/Latine	68.61%	66.00%	70.34%	70.30%	63.56%	58.33%
Race (%)						
White or Caucasian	68.11%	69.62%	64.33%	65.48%	62.50%	70.83%
Black or African American	14.02%	12.07%	17.20%	16.67%	16.18%	20.83%
Asian or Asian American	3.10%	2.41%	5.10%	5.95%	1.47%	0.00%
American Indian or Alaska Native	2.23%	3.22%	4.46%	3.57%	4.41%	0.00%
Native Hawaiian or Other Pacific Islander	0.25%	0.81%	0.00%	0.00%	0.74%	0.00%
Mixed race	8.31%	5.43%	5.73%	3.57%	9.56%	0.00%
Other/I wish not to respond	3.97%	6.44%	1.91%	4.76%	4.41%	8.33%

As Time 2 data were only used in the examination of test–retest reliability, the Time 2 samples above only describe those participants who provided usable data for both Time 1 and Time 2. Percentage of sample reporting Psychology as (one of) their college major(s) or minor(s): Sample 1 (54.0%), Sample 2 (49.7%), Sample 3 (53.6%), and Sample 4 (47.2%)

### Measures

#### Level of personality functioning scale-qIAT (LPFS-qIAT; introduced here)

The qIAT can be viewed as an amalgamation of self-report and IAT procedures that enables indirect measurement of a latent factor. Development of the LPFS-qIAT made use of the self-related logical categories presented by Friedman et al. [59] and items from the LPFS-BF 2.0 [25]. The qIAT requires opposing item sets; LPFS-BF 2.0 items reflect impaired personality functioning and a complementary set of items was created by reversing the meaning of the original LPFS-BF 2.0 items. The full list of LPFS-qIAT items is presented in Table 2. The LPFS-qIAT was built in R using the *iatgen* tool [60] with code modifications made to accommodate the qIAT method. The qIAT involves a procedure comparable to the IAT with minor differences. Specifically, participants complete multiple, brief double-categorization reaction-time tasks wherein a single statement (i.e., test item) is presented on the center of a computer screen along with category labels at the top right and top left corners of the screen. Participants are instructed to classify each statement using the presented category labels as quickly and as accurately as possible by pressing either the “E” or “I” key on their keyboard. The full procedure (see Table 3) consists of seven blocks, including two learning blocks (1, 2), three practice blocks (3, 5, 6), and two critical test blocks (4, 7). There are multiple strategies for handling incorrect responses (erroneous classifications), and the present project made use of two well-established methods for the purpose of generalizability: Sample 1 made use of a 600 ms error message with no response correction required and a forced error correction method was used with Samples 2, 3, and 4 [61]. A copy of the LPFS-qIAT for administration via Qualtrics and scoring code are available at https://osf.io/8bfka/?view_only=c1d5214c613547c1a07a0be548109d36. Details on data processing, scoring, and psychometric properties of the LPFS-qIAT are reported in the Results section below.

**Table 2:** LPFS-qIAT test stimuli

True	False
*Self-related logical categories and statements*	
– I am looking at a computer screen	– I am sunbathing at the beach
– I am participating in an experiment on the internet	– I am climbing a steep mountain
– I am doing a psychology experiment	– I am currently playing an electric guitar
– I am putting my fingers on the keyboard	– I am buying groceries in the local grocery store
– I am in front of the computer	– I am playing football on the grass

Impaired	Not impaired
*Personality functioning impairment categories and stimuli*	
– I often do not know who I really am	– I generally know who I really am
– I often think very negatively about myself	– I rarely think very negatively about myself
– My emotions change without me having a grip on them	– My emotions are usually well regulated and stable
– I have no sense of where I want to go in my life	– I have a good sense of where I want to go in my life
– I often do not understand my own thoughts and feelings	– I often understand my own thoughts and feelings
– I often make unrealistic demands of myself	– I often make realistic demands of myself
– I often have difficulty understanding the thoughts and feelings of others	– I often understand the thoughts and feelings of others
– I can't stand it when others have a different opinion than me	– I can appreciate others' opinions, even when we disagree
– I often don't understand the effect my behavior has on others	– I often understand the effect my behavior has on others
– My relationships and friendships usually don't last long	– My relationships and friendships usually last long
– I often feel uncomfortable when relationships become more intimate	– I often feel comfortable when relationships become more intimate
– I often have trouble cooperating with others	– I often cooperate with others well

**Table 3:** Task sequence for the LPFS-qIAT

Block	Trials	Task	Purpose	Left key	Right key
1	40	Discrimination of personality functioning impairment	Introduction to classification of personality functioning impairment and practice	Impaired	Not impaired
2	20	Discrimination of self-related logical statements	Introduction to classification of self-related logical statements and practice	True	False
3	20	Initial combined task (practice block)	Introduction to combined discrimination task and practice	Impaired or true	Not impaired or false
4	40	Initial combined task (test block)	Critical combined discrimination task	Impaired or true	Not impaired or false
5	40	Discrimination of personality functioning impairment-reversed	Practice classification of personality functioning impairment with keys reversed	Not impaired	Impaired
6	20	Reversed combined task (practice block)	Critical combined discrimination task with keys reversed	Not impaired or true	Impaired or false
7	40	Reversed combined task (test block)		Not impaired or true	Impaired or false

Response key designation and presentation of compatible or incompatible block were randomized in all studies. This example is one of four possible combinations

#### Level of personality functioning scale-brief form 2.0 (LPFS-BF 2.0)

The LPFS-BF 2.0 [25] is a 12-item self- and informant-rating measure of self-functioning and interpersonal functioning, producing two corresponding scale scores and a total personality functioning impairment score. This measure uses a 4-point Likert scale, ranging from 1 (*completely untrue*) to 4 (*completely true*). Internal consistencies were reported to be satisfactory for the total scale, as well as the self and interpersonal functioning scales, and meaningful associations were reported between the LPFS-BF 2.0 and other measures of severity of PDs [25]. The LPFS-BF 2.0’s total personality functioning impairment scale demonstrated acceptable internal consistency in the present studies for each sample, with reliability coefficients ranging from *α* = 0.88 and *ω* = 0.88 (Sample 1) to *α* = 0.90 and *ω* = 0.90 (Sample 4). Reliability coefficients of the informant-rating version were *α* = 0.88 and *ω* = 0.88 in both Sample 3 and Sample 4. It should be noted that the LPFS-BF 2.0 was replaced with the Level of Personality Functioning Scale-Self-Report (LPFS-SR) [26] for Samples 2 and 3. Study time constraints prevented administration of multiple self-report tests of personality functioning impairment and the authors opined that use of a criterion measure that does not share items with the LPFS-qIAT would make for a more robust scrutiny of the LPFS-qIAT’s validity. With respect to Sample 3 specifically, this replacement also allowed for a direct comparison of test–retest reliability results to previous external retest reliability findings (see [33], for a study on the retest reliability of the LPFS-SR). Due to the shorter length of the LPFS-BF 2.0, it replaced the LPFS-SR in the final study (Sample 4) to permit administration of a large assortment of measures used to examine the criterion and discriminant validity of the LPFS-qIAT.

#### Level of personality functioning scale-self report (LPFS-SR)

The LPFS-SR [26] is an 80-item self-report questionnaire designed to assess the severity of personality dysfunction by capturing the aspects of the LPFS in the AMPD. This measure uses a 4-point scale, ranging from *totally false* to *very true*. The LPFS-SR was reported to have good internal consistency and positive associations with other measures of similar constructs [26], as well as strong test–retest reliability over multiple weeks (*r*s = 0.81–0.91) [34]. The LPFS-SR total scale demonstrated acceptable internal consistency in each of the three studies including the LPFS-SR, with coefficients ranging from *α* = 0.94 and *ω* = 0.95 (Sample 3) to *α* = 0.95 and *ω* = 0.96 (Sample 2). Due to an already lengthy battery of tests being administered to Sample 4, the LPFS-SR was not included in the final study to avoid exhausting study participants.

#### DSM-5-TR self-rated level 1 cross-cutting symptom measure—adult (DSM-5-TR CC)

The DSM-5-TR CC [15] was developed to help clinicians assess all major areas of psychiatric functioning (e.g., mood, psychosis, cognition, personality, sleep) and identify additional areas for inquiry by revealing possible disorders, atypical presentations, subsyndromal conditions, and coexistent pathologies. The DSM-5-TR CC is endorsed by the American Psychiatric Association [15] as a necessary first step in identifying and addressing the heterogeneity of symptoms across diagnostic categories. The present study demonstrated acceptable internal consistency of the DSM-5-TR CC’s personality functioning domain scale (*α* = 0.80, *ω* = 0.80).

#### Personality inventory for DSM-5-brief form plus modified (PID5BF + M)

The PID5BF + M [62] is a 34-item self-report measure designed to assess the pathological trait domains described in Criterion B of the DSM-5’s AMPD and the chapter on personality disorders and related traits in the ICD-11: Negative Affectivity, Detachment, Disinhibition, Antagonism/Dissociality, Anankastia, and Psychoticism. The PID5BF + M is a modified version of the PID5BF + [63], which was derived from the Personality Inventory for DSM-5 [64] via ant colony optimization and demonstrated acceptable model fit, good reliability, and criterion-related validity across multiple samples [63]. The PID5BF + M was created to better capture the ICD-11 domain of anankastia and its three facets. The PID5BF + M has been shown to be a psychometrically sound instrument for assessing the six combined DSM-5 and ICD-11 personality trait domains [62]. Internal consistency reliability for the six trait domain scales used in the present studies ranged from *α* = 0.76 and *ω* = 0.76 (Detachment, Sample 3) to *α* = 0.88 and *ω* = 0.88 (Anankastia, Samples 3 and 4).

#### Schwartz outcome scale-10 (SOS-10)

The SOS-10 [65] is a 10-item self-report created to measure a broad domain of psychological health and well-being. Blais et al. [65] demonstrated the SOS-10’s convergent and divergent validity, ability to differentiate clinical and non-clinical samples, acceptable test–retest reliability, and good internal reliability (*α* = 0.96). The present study produced acceptable internal consistency of the SOS-10 (*α* = 0.94, *ω* = 0.94).

#### World Health Organization’s Quality of Life Brief Scale (WHOQOL-BREF)

The WHOQOL-BREF [66] is a 26-item self-report measure of four quality of life domains: Physical Health, Psychological, Social Relationships, and Environment. Derived from the WHOQOL-100, the WHOQOL-BREF was reported to correlate with WHOQOL-100 domain scores at approximately *r* = 0.90. Discriminant validity, content validity, and test–retest reliability were reported to be good for the WHOQOL-BREF [66]. The present study demonstrated acceptable internal consistency of domain scales, with reliability coefficients ranging from *α* = 0.67 and *ω* = 0.69 (Social Relationships) to *α* = 0.85 and *ω* = 0.85 (Psychological).

#### Inventory of depression and anxiety symptoms-second version (IDAS-II)

The IDAS-II [67] is a 99-item self-report questionnaire composed of 19 scales designed to assess a broad range of depression, anxiety, and bipolar symptoms. This measure uses a 5-point scale ranging from *not at all* to *extremely*. The IDAS-II scales have shown good convergent, discriminant, and criterion validity [67]. The present study demonstrated acceptable internal consistency with domain scale reliability coefficients of *α* = 0.80–0.91 and *ω* = 0.81–0.92.

#### Personality implicit association test-extraversion (extraversion IAT)

Using the methodology developed by Carpenter et al. [60], the extraversion IAT (see [68]) was administered via participants’ personal computers through an online data-collection platform (Qualtrics). Stimuli from the categories of self (*me, my, own, I, self*) and others (*they, your, them, you, others*) and items from the categories of extraversion (*sociable, talkative, active, impulsive, outgoing*) and introversion (*shy, withdrawn, passive, deliberate, reserved*) were presented [68]. Testing consisted of seven blocks and took approximately five minutes to complete. Relative to IATs measuring other self-concept constructs (e.g., racial or political biases), personality trait IATs have routinely demonstrated better reliability and construct validity; extraversion and neuroticism IATs appear to be the most robust [68]. Data processing procedures used for the Extraversion IAT are reported in the results section.

#### Social desirability-gamma short scale (KSE-G)

The KSE-G [69] is a 6-item self-report instrument that measures two aspects of socially desirable responding: Exaggeration of positive qualities (PQ+) and Minimization of negative qualities (NQ−). Originally developed in German (Kurzskala Soziale Erwünschtheit–Gamma) [70], the English-language adaptation has demonstrated reliability and validity coefficients comparable to those of the original German version, as well as metric measurement invariance [69]. Convergent and discriminant validity of the KSE-G supports the instrument’s construct validity, and estimates of internal consistency (*α*) have ranged between 0.65 and 0.72 for PQ + and between 0.64 and 0.79 for NQ− [69]. The present study obtained approximately comparable levels of internal consistency for the PQ + (*α* = 0.58, *ω* = 0.58) and the NQ− (*α* = 0.65, *ω* = 0.65).

#### Indecisiveness Scale (IS)

The IS [71] is a 22-item self-report measure of general indecisiveness, which pertains to difficulties in making decisions regardless of whether they are of great or little significance. The IS is composed of 11 features, each of which include one positively formulated and one negatively formulated item. Psychometric study of the IS has found support for a unifactor structure, acceptable levels of test–retest reliability, and predictive validity in terms of various decision-making situations [71]. The Present study demonstrated acceptable internal consistency (*α* = 0.92, *ω* = 0.92).

### Statistical analyses

Following development of the LPFS-qIAT, four studies were conducted to examine the LPFS-qIAT’s internal and retest reliability and the convergent, discriminant, and criterion-related validity of this new instrument. The LPFS-qIAT and Extraversion IAT data for all samples were processed using a *D*-score data cleaning and scoring algorithm (see [60]). First, participants with more than 10 percent of responses faster than 300 ms (i.e., excessively fast responders) were removed. Then, in Sample 1, participant response errors were addressed by replacing participants’ response errors with their block means of correct trials plus 600 ms (i.e., the *D*_600_ procedure). The built-in-error-penalty method, wherein participants are required to self-correct an error before proceeding, is a preferred method [61, 72] and was used in Samples 2, 3, and 4. The use of different methods across studies was done for the purpose of generalizability. Finally, LPFS-qIAT and Extraversion IAT responses were *D*-scored, with positive scores indicating greater levels of personality functioning impairment or extraversion. Internal consistency reliability of the LPFS-qIAT was assessed using a variant of Cronbach’s alpha [73], and Pearson correlations using a bootstrapping procedure with 5000 replicates were used to evaluate the instrument’s test–retest reliability. Correlation coefficients and associated 95% confidence intervals based on 5000 bootstrap replicates were also calculated to evaluate the convergent, discriminant, and criterion-related validity of the LPFS-qIAT. In all cases, the statistical significance of an effect size was determined by examining the 95% confidence intervals computed around the estimated effect size and deemed significant at *p* < 0.05 when a value of zero (null) did not fall between the upper and lower intervals.

## Results

### Data processing

For all samples, the LPFS-qIAT and Extraversion IAT data were processed using a *D*-score data cleaning and scoring algorithm (see [60]). The percent of dropped trials (trials > 10 s) was low, ranging from 0.0043% (Time 2 Sample 4) to 0.0057% (Time 1 Sample 4). The percentage of participants removed due to excessively fast responding, which was expected to be elevated in our student convenience samples, ranged from 22.7% (Sample 1) to 29.8% (Sample 2) and was within the ranges commonly seen for anonymous online data collections [60]. As further detailed in the Supplementary Material, removed participants (i.e., excessively fast responders) were significantly younger and self-reported higher levels of psychopathology than retained participants in our two large samples (Samples 1 and 2). This pattern was not observed in Samples 3 or 4, nor were removed participants in Samples 3 and 4 found to differ from retained participants with respect to informant ratings. Participants in Samples 3 and 4 who completed Time 2 did not significantly differ from those who dropped out on any measure of personality functioning impairment, but drop-out participants did endorse significantly higher levels of disinhibition and psychoticism at Time 1.

Error rates on the LPFS-qIAT ranged from 11.4% (Sample 2) to 14.3% (Time 1 Sample 3) and were handled using two different methods across studies. For Sample 1, participants’ response errors were replaced with their block means of correct trials plus 600 ms (i.e., the *D*_600_ procedure). The built-in-error-penalty method, wherein participants are required to self-correct an error before proceeding, is a preferred method [61, 72] and was used in Samples 2, 3, and 4. The use of different methods across studies was done for the purpose of generalizability. LPFS-qIAT and Extraversion IAT responses were then *D*-scored, with positive scores indicating greater levels of personality functioning impairment or extraversion.

### Reliability

#### Internal consistency reliability

Internal consistency reliability of the LPFS-qIAT, assessed using a variant of Cronbach’s alpha [73], was found to approach, and in most cases, exceed that typically observed for the IAT (averaged *α* = 0.80) [47]. Except for Sample 2 (*α* = 0.792) and Sample 3 Time 2 (*α* = 0.792), internal consistency reliability of the LPFS-qIAT was above 0.800 and ranged from 0.820 to 0.875.

#### Test–retest reliability

One hundred four participants from Sample 3 and 48 participants from Sample 4 completed the LPFS-qIAT at both Time 1 and Time 2 of their respective studies and were used to calculate test–retest reliability over the span of one and two weeks, respectively. Pearson correlations using a bootstrapping procedure with 5000 replicates were used to evaluate test–retest reliability. Calculations produced a correlation coefficient of 0.381 (*n* = 104; 95% CI 0.200, 0.552) for test–retest reliability over one week and a coefficient of 0.478 (*n* = 48, 95% CI 0.285, 0.661) over two weeks. Surprisingly, the LPFS-qIAT demonstrated slightly better test–retest reliability over a one-week period than the LPFS-SR in the same study (*r* = 0.334; *n* = 102; 95% CI 0.137, 0.516), although this was substantially poorer than that found by Hopwood et al. [33] for the same test over a two-week period (*r*s = 0.81–0.91). The test–retest reliability of the LPFS-qIAT over a two-week period was poorer than that found for the LPFS-BF 2.0 over the same duration (*r* = 0.658; *n* = 46; 95% CI 0.386, 0.910). To our knowledge, the test–retest reliability of the LPFS-BF 2.0 has not been published or investigated, making this a novel finding.

### Validity

#### Convergent and criterion-related validity

Convergent validity of the LPFS-qIAT with self-report measures of personality functioning impairment was evaluated in each of the four samples, as well as with informant-rating measures in Samples 3 and 4. Correlation coefficients and associated 95% confidence intervals based on 5000 bootstrap replicates are reported in Table 4. Overall, the LPFS-qIAT significantly correlated with all self-report and informant-rating measures of personality functioning impairment in the expected direction. Correlation coefficients ranged from *r* = 0.143 (95% CI 0.006, 0.282) (LPFS-SR in Sample 3) to *r* = 0.227 (95% CI 0.090, 0.362) (LPFS-BF 2.0 in Sample 4), with all correlations significant and most correlations hovering around the *r* = 0.20 range.^3^ The obtained effect sizes are comparable to those typically observed between performance-based and self-report or informant-rating scores on measures of analogous constructs (typically in the *r* = 0.20–0.30 range) [53–56].

**Table 4:** Correlation coefficients depicting the convergent validity of the LPFS-qIAT with other measures of personality functioning impairment and the LPFS-qIAT’s criterion-related validity

Measure	Method	Sample 1	Sample 2	Sample 3	Sample 4
		(*N* = 806)	(*N* = 497)	(*N* = 209)	(*N* = 236)
lpfs-bf 2.0 total	Self-report	**0.194** (0.128, 0.261)	–	–	**0.227** (0.090, 0.362)
	Informant^a^	–	–	**0.148** (0.051, 0.244)	
LPFS-SR total	Self-report	**0.189** (0.118, 0.257)	**0.198** (0.112, 0.292)	**0.143** (0.006, 0.282)	–
DSM-5-TR CC PF	Self-report	–	–	–	**0.187** (0.054, 0.313)
Negative affectivity	Self-report	**0.124** (0.054, 0.191)	**0.097** (0.011, 0.182)	**0.139** (0.016, 0.261)	**0.138** (0.020, 0.251)
Detachment	Self-report	**0.163** (0.097, 0.229)	**0.154** (0.066, 0.243)	0.059 (− 0.068, 0.190)	**0.207** (0.080, 0.329)
Antagonism	Self-report	**0.089** (0.019, 0.160)	0.027 (− 0.053, 0.109)	0.049 (− 0.092, 0.193)	0.090 (− 0.052, 0.222)
Disinhibition	Self-report	**0.124** (0.053, 0.193)	**0.114** (0.020, 0.207)	0.065 (− 0.067, 0.192)	**0.142** (0.004, 0.272)
Anankastia	Self-report	**0.080** (0.014, 0.142)	0.081 (− 0.006, 0.169)	0.016 (− 0.119, 0.155)	− 0.010 (− 0.134, 0.113)
Psychoticism	Self-report	**0.082** (0.012, 0.150)	**0.091** (0.005, 0.177)	0.038 (− 0.087, 0.164)	0.113 (− 0.025, 0.244)
SOS-10	Self-report	–	–	–	**− 0.131** (− 0.003, − 0.254)
WHOQOL-BREF physical	Self-report	–	–	–	− 0.123 (0.006, − 0.250)
WHOQOL-BREF psychological	Self-report	–	–	–	− 0.118 (0.017, − 0.247)
WHOQOL-BREF social relationships	Self-report	–	–	–	**− 0.137** (− 0.007, − 0.263)
WHOQOL-BREF environment	Self-report	–	–	–	**− 0.150** (− 0.034, − 0.266)

Bold indicates statistical significance based on 95% confidence intervals from 5000 bootstrap replicates. Sample 1 personality traits scores were obtained using the PID5BF + [62] whereas trait scores for all other samples obtained using the PID5BF + M [61]

^a^412 informant ratings of 285 participants

Also reported in Table 4 are results pertaining to the LPFS-qIAT’s criterion-related validity. LPFS-qIAT scores were significantly positively related to Negative Affectivity in all samples, and occasionally associated with other maladaptive personality trait scores; however, correlations between the LPFS-qIAT and trait scores were consistently smaller in magnitude than those observed between the LPFS-qIAT and measures of personality functioning impairment. The LPFS-qIAT was significantly negatively correlated with overall psychological health (SOS-10), quality of social relationships (WHOQOL-BREF Social Relationships), and perceived environmental quality and resources (WHOQOL-BREF Environment). Associations between LPFS-qIAT scores and psychological (*r* = − 0.118; 95% CI 0.017, − 0.247) and physical (*r* = -0.123; 95% CI 0.006, − 0.250) health were in the expected direction but did not reach a level of statistical significance at *p* < 0.05.

#### Discriminant validity

Discriminant validity of the LPFS-qIAT was investigated through its association with self-report measures of depression, anxiety, indecisiveness, socially desirable responding, and age as well as its association with an IAT measure of extraversion. Sensitivity analysis based on sample size for a two-tailed significance test given α = 0.05 and power of 0.80 indicated minimum detectable effect sizes ranging from *r* = 0.098 (Sample 1) to *r* = 0.192 (Sample 3), evidencing that the present study’s discriminant validity analysis was powerful enough to detect even small effect sizes. Correlation coefficients and associated 95% CIs based on 5000 bootstrap replicates are reported in Table 5. In all cases, LPFS-qIAT scores minimally and non-significantly correlated with scores representing theoretically unrelated (or minimally related) constructs. Importantly, all effect sizes were smaller than those observed between the LPFS-qIAT and all self-report and informant-rating measures of personality functioning impairment.

**Table 5:** Correlation coefficients depicting the discriminant validity of the LPFS qIAT

Discriminant measure	Sample 1	Sample 2	Sample 3	Sample 4
	(*N* = 806)	(*N* = 497)	(*N* = 209)	(*N* = 236)
Age	− 0.057 (− 0.133, 0.021)	− 0.040 (− 0.117, 0.039)	− 0.095 (− 0.234, 0.046)	− 0.083 (− 0.178, 0.026)
Extraversion IAT	–	0.005 (− 0.085, 0.091)	–	–
IDAS-II				
General depression	–	–	–	0.087 (− 0.049, 0.214)
Dysphoria	–	–	–	0.085 (− 0.049, 0.213)
Lassitude	–	–	–	0.095 (− 0.042, 0.226)
Insomnia	–	–	–	0.040 (− 0.088, 0.166)
Suicidality	–	–	–	− 0.037 (− 0.170, 0.104)
Appetite loss	–	–	–	0.014 (− 0.118, 0.142)
Appetite gain	–	–	–	0.079 (− 0.048, 0.203)
Well-being	–	–	–	− 0.107 (− 0.240, 0.032)
Ill temper	–	–	–	0.054 (− 0.085, 0.195)
Mania	–	–	–	0.066 (− 0.068, 0.197)
Euphoria	–	–	–	0.057 (− 0.064, 0.177)
Panic	–	–	–	0.044 (− 0.089, 0.176)
Social anxiety	–	–	–	0.081 (− 0.048, 0.209)
Claustrophobia	–	–	–	0.046 (− 0.092, 0.181)
Traumatic intrusions	–	–	–	0.023 (− 0.122, 0.169)
Traumatic avoidance	–	–	–	0.091 (− 0.049, 0.230)
Checking	–	–	–	0.045 (− 0.080, 0.164)
Ordering	–	–	–	0.056 (− 0.074, 0.185)
Cleaning	–	–	–	0.053 (− 0.081, 0.185)
KSE-G				
Exaggerating positive qualities	–	–	–	− 0.024 (− 0.150, 0.101)
Minimizing negative qualities	–	–	–	0.063 (− 0.082, 0.200)
Indecisiveness Scale				
Total indecisiveness	–	–	–	− 0.019 (− 0.161, 0.126)
Minimum detectable effect size	0.098	0.127	0.192	0.181

All correlation coefficients were found to be non-significant based on 95% confidence intervals from 5000 bootstrap replicates. Minimum detectable effect sizes derived from sensitivity analysis based on sample size and two-tailed significance test given α = 0.05 and power of 0.80

## Discussion

Available measures of personality functioning impairment as operationalized in the AMPD and ICD-11 exclusively use self-report, clinician-/informant-rated, or interview methods and there is a problematic absence of performance-based instruments capable of measuring personality functioning impairment. The LPFS-qIAT takes a step in filling this gap as the first performance-based measure of personality functioning impairment that directly parallels the AMPD and, to a degree, the ICD-11. The LPFS-qIAT is brief, with completion taking 5–10 min, is easily administered in person or online, and can be automatically scored in a manner allowing simple interpretation. This initial psychometric evaluation of the LPFS-qIAT produced encouraging evidence across four separate studies.

The LPFS-qIAT’s good internal reliability was repeatedly observed, demonstrating internal consistency for this specific instrument, and allowing confidence in its online administration. The latter is a notable strength of the LPFS-qIAT compared to most other performance-based measures of personality. Although poor by self-report standards, one- and two-week test–retest reliability coefficients were approaching those often seen for IAT measures (averaged *r* = 0.50) [47], but are at levels insufficient to justify interpreting a single individual’s score. Convergent validity of the LPFS-qIAT was established using three different self-report tests of personality functioning impairment and one informant-rated measure. The correlation coefficients obtained across the four studies were comparable in magnitude to those normally observed between performance-based (e.g., Rorschach, TAT) and self-report or informant-rating measures of analogous constructs (typically in the *r* = 0.20–0.30 range) [53–56]. Evidence of the LPFS-qIAT’s criterion-related validity was demonstrated by its positive correlation with maladaptive personality traits, particularly Negative Affectivity, and inverse relationships with measures of psychological health and quality of life. Finally, discriminant validity of the LPFS-qIAT was exhibited through small, non-significant correlations with self-report measures of depression, anxiety, indecisiveness, socially desirable responding, and age as well as a negligible and non-significant association with an IAT measure of extraversion. Taken together, the LPFS-qIAT can be cautiously viewed as an internally reliable and valid measure of personality functioning impairment based on this initial, but rather extensive psychometric examination. The LPFS-qIAT’s test–retest reliability emerged as a notable limitation that must be addressed in future studies.

### Limitations, constraints on Generalizability, and future directions

Although encouraging, the present studies are not without their limitations. First, each of the reported studies were conducted using diverse college student convenience samples, limiting the generalizability of findings. The present samples are more diverse in race and ethnicity than common for college student samples, but the current findings might not generalize to a broader population. Future studies should build upon the somewhat promising findings reported herein through generalizability studies using more diverse samples, including both community and clinical populations. Similarly, differences between retained/continued and removed/dropout participants may also limit the generalizability of results and should be investigated in future studies. However, it is necessary to first address the limitations of the LPFS-qIAT’s retest reliability. The poor retest reliability results were obtained via online studies using small convenience samples. Future research may wish to first investigate the LPFS-qIAT’s test–retest reliability in larger, more reliable samples. Should reliability coefficients not meaningfully improve, researchers should explore tactics for increasing retest reliability to a level that would allow confidence. One prospective solution to this issue involves averaging results from a repeated administration of the LPFS-qIAT at a single timepoint, a strategy often successfully used in research and clinical practice for other measures (e.g., blood pressure) [74]. Encouragingly, researchers using this strategy recently produced an IAT test–retest reliability of *r* = 0.89 over a period of 2 years [75]. The current study also did not specifically evaluate the LPFS-qIAT’s incremental validity over other methods, such as self-report measures, when predicting meaningful outcomes (e.g., actual problems in relationships or at work). Investigating this instrument’s incremental validity in future studies is important. When doing so, researchers must be mindful of undesired method effects and not measure outcomes using the same method against which the LPFS-qIAT is being compared (e.g., outcomes measured by self-reports should not be used when the LPFS-qIAT’s incremental validity over self-report measures of personality functioning is being tested). Lastly, the LPFS-qIAT possibly fails to account for all components of the ICD-11’s conceptualization of personality functioning, which is represented by self- and interpersonal dysfunction *in addition to* emotional, cognitive, and behavioral manifestations of the personality dysfunction [13]. Future studies will need to investigate the degree to which the LPFS-qIAT can capture all components of personality functioning impairment enumerated in the ICD-11. That is, future studies should test the LPFS-qIAT’s construct validity using ICD-11-compliant criterion measures. Should the LPFS-qIAT demonstrate poor construct validity in those studies, researchers might choose to apply the methodology described in this paper to a self-report measure that directly aligns with the ICD-11, such as the PDS-ICD-11 [31], to develop a new performance-based instrument that more closely matches the ICD-11’s conceptualization of personality functioning. Despite these limitations and need for further studies, development (and further validation) of a novel, easily administered and automatically scored performance-based measure of personality functioning impairment has several implications.

Professional practice guidelines advocate for the use of multimethod assessment [45, 76, 77]. The availability of a performance-based measure of personality functioning impairment that is psychometrically promising, aligns with the AMPD (and partly with the ICD-11), and is easily administered, scored, and interpreted can prove valuable. Multimethod research designs minimize unwanted measurement effects, and the examination of convergences and divergences of test scores derived from multiple methods for measuring analogous constructs—intertest score relationships—can facilitate a more comprehensive and precise measurement of study variables. When similar strategies are applied in clinical practice, clinicians can gain a greater understanding of the relationship between a client’s explicit and implicit personality dysfunction. Hence, the LPFS-qIAT, as the first performance-based measure of personality functioning aligning with contemporary models, holds potential to become a useful tool in both research and clinical practice by facilitating multimethod assessment. However, researchers must first address the LPFS-qIAT’s poor retest reliability, investigate the generalizability of the present findings, and evaluate this instrument’s incremental validity over other methods when predicting meaningful outcomes before this statement can be made more definitively. The LPFS-qIAT might also benefit research and clinical practice as a novel method for capturing changes in personality dysfunction. The LPFS-qIAT was derived from the LPFS-BF 2.0, and the latter has shown a high sensitivity to change in response to therapy [25]. Should future studies find that the LPFS-qIAT can also detect therapy-related changes in personality functioning, the LPFS-qIAT could be a useful tool for therapists and in therapy outcome research—perhaps especially when paired with the LPFS-BF 2.0. Alternatively, scores derived from performance-based measures such as the LPFS-qIAT are largely based on unrehearsed performance, which could interfere with using the instrument in this way due to the potential for practice effects [78]. The effect of repeated measurement using the LPFS-qIAT over the course of treatment, the potential measurement error due to practice effects, and the instrument’s validity and utility for therapy outcome research could be important questions to explore once the other psychometric needs noted above have been met (e.g., improved retest reliability). Nevertheless, the findings of the reported studies offer encouragement for future research and continuing to improve and validate the LPFS-qIAT as a robust, performance-based measure of personality functioning.

### Supplementary Information

Below is the link to the electronic supplementary material.

Supplementary file1 (DOCX 96 KB)

## Data Availability

Deidentified data are only available upon reasonable request made to the first author (per IRB guidelines). Copies of the LPFS-qIAT and analysis code can be found in our online supplementary documents (see https://osf.io/8bfka/). This study was not preregistered.

## Footnotes

1 Although not an investigation of test–retest reliability (temporal stability), it is worth mentioning here that Weekers et al. [25] demonstrated the LPFS-BF 2.0’s ability to detect expected changes over time in response to treatment by administering the instrument to treatment-seeking adults at intake and again at the end of treatment, with a mean time between administrations of 92.13 days. In doing so, they also found a pre-post correlation of* r* = 0.49.

2 Although poor by most standards, test–retest reliability coefficients of *r* = 0.50 can be considered adequate for use in correlational and group differences research but would be insufficient to justify interpreting a single individual’s score.

3 As shown in Table S16 of the Supplementary Material, correlations between the LPFS-qIAT and other measures of personality functioning impairment remained statistically significant when participants that had been removed for excessively fast responding were reintroduced. These results help minimize concerns over the statistically significant differences seen on some measures of psychopathology between participants who were retained and those who were removed in Samples 1 and 2.
