# Supplementary material for: A new performance-based measure of personality functioning impairment: development and preliminary evaluation of reliability and validity
Source: Discov Ment Health. 2024 Feb 22;4(1):6. doi: 10.1007/s44192-024-00059-4 (PMC10884381; doi:10.1007/s44192-024-00059-4)
Supplement: Supplementary file 1 — Supplementary file1 (DOCX 96 KB) [file 44192_2024_59_MOESM1_ESM.docx]

A New Performance-Based Measure of Personality Functioning Impairment: Development and Evaluation of Reliability and Validity

**SUPPLEMENTARY MATERIAL**

**Attrition and Participant Removal Group Comparisons**

| Table S1: *Descriptive Statistics of Sample 1 Groups* | | | |
| --- | --- | --- | --- |
|  | | Retained | Removed |
|  |  | (*N* = 806) | (*N* = 237) |
| Age, μ (SD) | | 21.19 (4.83) | 20.19 (3.11) |
| Sex (%) | |  |  |
|  | Female | 82.88% | 81.01% |
|  | Male | 16.50% | 15.61% |
|  | Other / I wish not to respond | 0.50% | 0.42% |
|  | Missing | 0.12% | 2.95% |
| Ethnicity (%) | |  |  |
|  | Hispanic / Latine | 31.39% | 31.65%% |
|  | Non-Hispanic / Latine | 68.49% | 65.40%% |
|  | Missing | 0.12% | 2.95% |
| Race (%) | |  |  |
|  | White or Caucasian | 68.11% | 50.63% |
|  | Black or African American | 14.02% | 29.11% |
|  | Asian or Asian American | 3.10% | 1.69% |
|  | American Indian or Alaska Native | 2.23% | 2.11% |
|  | Native Hawaiian or Other Pacific Islander | 0.25% | 0.42% |
|  | mixed race | 8.31% | 7.60% |
|  | other / I wish not to respond | 3.97% | 4.64% |
|  | Missing | 1.24% | 3.80% |
|  | | | |

| Table S2: *Group Comparisons of Sample 1* | | | | | | | |
| --- | --- | --- | --- | --- | --- | --- | --- |
|  | Retained | | Removed | |  |  |  |
|  | *M* | *SE* | *M* | *SE* | *t* | *p* |  |
| Age | 21.19 | 0.17 | 20.19 | 0.21 | -3.756 | < .001 |  |
| LPFS-BF 2.0 Total | 24.58 | 0.25 | 25.73 | 0.54 | 1.931 | .054 |  |
| LPFS-SR Total | 267.58 | 2.41 | 304.69 | 5.56 | 6.122 | < .001 |  |
| PID5BF+ Negative Affectivity | 14.44 | 0.16 | 14.05 | 0.29 | -1.162 | .246 |  |
| PID5BF+ Detachment | 11.13 | 0.13 | 12.50 | 0.28 | 4.431 | < .001 |  |
| PID5BF+ Antagonism | 10.17 | 0.12 | 12.42 | 0.29 | 7.188 | < .001 |  |
| PID5BF+ Disinhibition | 11.87 | 0.14 | 12.96 | 0.28 | 3.515 | < .001 |  |
| PID5BF+ Anankastia | 8.00 | 0.09 | 8.67 | 0.19 | 3.116 | .002 |  |
| PID5BF+ Psychoticism | 11.99 | 0.15 | 12.73 | 0.29 | 2.260 | .024 |  |

| Table S3: *Descriptive Statistics of Sample 2 Groups* | | | |
| --- | --- | --- | --- |
|  | | Retained | Removed |
|  |  | (*N* = 497) | (*N* = 211) |
| Age, μ (SD) | | 21.12 (4.91) | 20.18 (3.31) |
| Sex (%) | |  |  |
|  | Female | 85.71% | 77.25% |
|  | Male | 13.88% | 22.28% |
|  | Other / I wish not to respond | 0.20% | 0.00% |
|  | Missing | 0.20% | 0.47% |
| Ethnicity (%) | |  |  |
|  | Hispanic / Latine | 34.00% | 38.86% |
|  | Non-Hispanic / Latine | 65.59% | 60.19% |
|  | Missing | 0.40% | 0.95% |
| Race (%) | |  |  |
|  | White or Caucasian | 69.62% | 61.14% |
|  | Black or African American | 12.07% | 22.28% |
|  | Asian or Asian American | 2.41% | 1.42% |
|  | American Indian or Alaska Native | 3.22% | 3.32% |
|  | Native Hawaiian or Other Pacific Islander | 0.81% | 0.47% |
|  | mixed race | 5.43% | 5.69% |
|  | other / I wish not to respond | 5.43% | 4.74% |
|  | Missing | 1.01% | 0.95% |
|  | | | |

| Table S4: *Group Comparisons of Sample 2* | | | | | | | |
| --- | --- | --- | --- | --- | --- | --- | --- |
|  | Retained | | Removed | |  |  |  |
|  | *M* | *SE* | *M* | *SE* | *t* | *p* |  |
| Age | 21.12 | 0.22 | 20.18 | 0.29 | -2.943 | .003 |  |
| LPFS-SR Total | 265.56 | 3.17 | 294.97 | 5.76 | 4.463 | < .001 |  |
| PID5BF+M Negative Affectivity | 5.00 | 0.07 | 4.64 | 0.10 | -2.998 | .003 |  |
| PID5BF+M Detachment | 3.75 | 0.05 | 4.36 | 0.10 | 5.400 | < .001 |  |
| PID5BF+M Antagonism | 3.47 | 0.05 | 4.21 | 0.10 | 6.822 | < .001 |  |
| PID5BF+M Disinhibition | 4.17 | 0.06 | 4.48 | 0.09 | 2.905 | .004 |  |
| PID5BF+M Anankastia | 4.30 | 0.07 | 4.53 | 0.10 | 1.997 | .046 |  |
| PID5BF+M Psychoticism | 4.10 | 0.07 | 4.50 | 0.10 | 3.371 | < .001 |  |

| Table S5: *Descriptive Statistics of Sample 3 Groups* | | | | | |  |  |
| --- | --- | --- | --- | --- | --- | --- | --- |
|  | | Retained | Removed | Continued | Dropout | Retained  Continued | Retained  Dropout |
|  |  | (*N* = 209) | (*N* = 78) | (*N* = 138) | (*N* = 149) | (*N* = 118) | (*N* = 91) |
| Age, μ (SD) | | 21.81 (6.86) | 20.26 (2.91) | 21.64 (7.30) | 21.17 (4.78) | 21.84 (7.74) | 21.78 (5.62) |
| Sex (%) | |  |  |  |  |  |  |
|  | Female | 87.08% | 84.62% | 86.96% | 85.91% | 87.29% | 86.81% |
|  | Male | 12.44% | 15.39% | 12.32% | 14.09% | 11.86% | 13.19% |
|  | Other / I wish not to respond | 0.48% | 0.00% | 0.73% | 0.00% | 0.85% | 0.00% |
|  | Missing | 0.00% | 0.00% | 0.00% | 0.00% | 0.00% | 0.00% |
| Ethnicity (%) | |  |  |  |  |  |  |
|  | Hispanic / Latine | 29.19% | 26.92% | 31.88% | 25.50% | 31.36% | 26.37% |
|  | Non-Hispanic / Latine | 70.81% | 70.51% | 68.12% | 73.15% | 68.64% | 73.63% |
|  | Missing | 0.00% | 2.56% | 0.00% | 1.34% | 0.00% | 0.00% |
| Race (%) | |  |  |  |  |  |  |
|  | White or Caucasian | 65.55% | 56.41% | 65.22% | 61.07% | 65.25% | 65.93% |
|  | Black or African American | 16.75% | 32.05% | 16.67% | 24.83% | 15.25% | 18.68% |
|  | Asian or Asian American | 4.79% | 3.85% | 3.62% | 5.37% | 4.24% | 5.50% |
|  | American Indian or Alaska Native | 3.83% | 1.28% | 3.62% | 2.69% | 4.24% | 3.30% |
|  | Native Hawaiian or Other Pacific Islander | 0.00% | 1.28% | 0.00% | 0.67% | 0.00% | 0.00% |
|  | mixed race | 4.79% | 2.56% | 6.52% | 2.01% | 6.78% | 2.20% |
|  | other / I wish not to respond | 3.35% | 2.56% | 2.90% | 3.36% | 2.54% | 4.40% |
|  | Missing | 0.96% | 0.00% | 1.45% | 0.00% | 1.70% | 0.00% |
|  | | | | | |  |  |

| Table S6: *Group Comparisons of Sample 3* | | | | | | | |
| --- | --- | --- | --- | --- | --- | --- | --- |
|  | Retained | | Removed | |  |  |  |
|  | *M* | *SE* | *M* | *SE* | *t* | *p* |  |
| Age | 21.81 | 0.48 | 20.26 | 0.33 | -2.678 | .008 |  |
| LPFS-SR Total | 162.11 | 9.24 | 177.89 | 16.87 | 0.820 | .414 |  |
| PID5BF+M Negative Affectivity | 4.67 | 0.10 | 4.45 | 0.14 | -1.287 | .200 |  |
| PID5BF+M Detachment | 3.85 | 0.09 | 4.02 | 0.14 | 1.010 | .314 |  |
| PID5BF+M Antagonism | 3.48 | 0.10 | 3.92 | 0.16 | 2.389 | .018 |  |
| PID5BF+M Disinhibition | 3.84 | 0.09 | 3.97 | 0.16 | 0.662 | .509 |  |
| PID5BF+M Anankastia | 3.98 | 0.11 | 3.95 | 0.17 | -0.156 | .876 |  |
| PID5BF+M Psychoticism | 3.95 | 0.11 | 4.24 | 0.16 | 1.502 | .135 |  |

| Table S7: *Group Comparisons of Sample 3* | | | | | | | |
| --- | --- | --- | --- | --- | --- | --- | --- |
|  | Continued | | Dropout | |  |  |  |
|  | *M* | *SE* | *M* | *SE* | *t* | *p* |  |
| Age | 21.64 | 0.62 | 21.17 | 0.39 | -0.645 | .520 |  |
| LPFS-qIAT | -0.31 | 0.04 | -0.32 | 0.04 | -0.120 | .905 |  |
| LPFS-SR Total | 161.99 | 11.31 | 170.40 | 11.68 | 0.518 | .605 |  |
| PID5BF+M Negative Affectivity | 4.62 | 0.12 | 4.61 | 0.12 | -0.063 | .950 |  |
| PID5BF+M Detachment | 3.86 | 0.11 | 3.93 | 0.11 | 0.450 | .653 |  |
| PID5BF+M Antagonism | 3.46 | 0.19 | 3.74 | 0.12 | 1.638 | .103 |  |
| PID5BF+M Disinhibition | 3.80 | 0.11 | 3.95 | 0.12 | 0.896 | .371 |  |
| PID5BF+M Anankastia | 3.85 | 0.13 | 4.09 | 0.13 | 1.316 | .189 |  |
| PID5BF+M Psychoticism | 3.94 | 0.13 | 4.12 | 0.12 | 0.986 | .325 |  |

| Table S8: *Group Comparisons of Sample 3* | | | | | | | |
| --- | --- | --- | --- | --- | --- | --- | --- |
|  | Retained Continued | | Retained Dropout | |  |  |  |
|  | *M* | *SE* | *M* | *SE* | *t* | *p* |  |
| Age | 21.84 | 0.71 | 21.78 | 0.59 | -0.064 | .949 |  |
| LPFS-SR Total | 160.97 | 12.05 | 163.64 | 14.46 | -0.120 | .905 |  |
| PID5BF+M Negative Affectivity | 4.70 | 0.14 | 4.64 | 0.16 | 0.142 | .887 |  |
| PID5BF+M Detachment | 3.84 | 0.12 | 3.88 | 0.14 | 0.212 | .832 |  |
| PID5BF+M Antagonism | 3.39 | 0.13 | 3.60 | 0.15 | 1.024 | .307 |  |
| PID5BF+M Disinhibition | 3.81 | 0.12 | 3.89 | 0.15 | 0.435 | .664 |  |
| PID5BF+M Anankastia | 3.91 | 0.14 | 4.08 | 0.17 | 0.738 | .462 |  |
| PID5BF+M Psychoticism | 3.90 | 0.14 | 4.03 | 0.16 | 0.618 | .537 |  |

| Table S9: *Group Comparisons of Sample 3 Informant Ratings* | | | | | | | |
| --- | --- | --- | --- | --- | --- | --- | --- |
|  | Retained | | Removed | |  |  |  |
|  | *M* | *SE* | *M* | *SE* | *t* | *p* |  |
| LPFS-BF 2.0 Total | 20.24 | 0.46 | 20.82 | 1.09 | 0.492 | .625 |  |
| PID5BF+M Negative Affectivity | 4.30 | 0.11 | 4.26 | 0.24 | -0.156 | .879 |  |
| PID5BF+M Detachment | 3.20 | 0.07 | 3.46 | 0.19 | 1.306 | .198 |  |
| PID5BF+M Antagonism | 2.59 | 0.06 | 3.03 | 0.20 | 2.098 | .041 |  |
| PID5BF+M Disinhibition | 3.28 | 0.07 | 3.52 | 0.20 | 1.164 | .250 |  |
| PID5BF+M Anankastia | 4.34 | 0.11 | 4.18 | 0.27 | -0.534 | .596 |  |
| PID5BF+M Psychoticism | 3.22 | 0.08 | 3.27 | 0.20 | 0.232 | .818 |  |

| Table S10: *Descriptive Statistics of Sample 4 Groups* | | | | | |  |  |
| --- | --- | --- | --- | --- | --- | --- | --- |
|  | | Retained | Removed | Continued | Dropout | Retained  Continued | Retained  Dropout |
|  |  | (*N* = 236) | (*N* = 84) | (*N* = 74) | (*N* = 246) | (*N* = 58) | (*N* = 178) |
| Age, μ (SD) | | 20.60 (4.58) | 20.25 (3.28) | 20.85 (5.47) | 20.41 (3.87) | 21.33 (6.03) | 20.37 (3.99) |
| Sex (%) | |  |  |  |  |  |  |
|  | Female | 86.44% | 79.76% | 86.49% | 84.15% | 87.93% | 85.96% |
|  | Male | 12.71% | 19.05% | 12.16% | 15.04% | 12.07% | 12.92% |
|  | Other / I wish not to respond | 0.85% | 0.00% | 0.00% | 0.81% | 0.00% | 1.12% |
|  | Missing | 0.00% | 1.19% | 1.35% | 0.00% | 0.00% | 0.00% |
| Ethnicity (%) | |  |  |  |  |  |  |
|  | Hispanic / Latine | 36.44% | 23.81% | 43.24% | 30.08% | 48.28% | 32.58% |
|  | Non-Hispanic / Latine | 63.56% | 75.00% | 55.41% | 69.92% | 51.72% | 67.42% |
|  | Missing | 0.00% | 1.19% | 1.35% | 0.00% | 0.00% | 0.00% |
| Race (%) | |  |  |  |  |  |  |
|  | White or Caucasian | 66.53% | 52.38% | 67.57% | 61.38% | 68.97% | 65.73% |
|  | Black or African American | 13.56% | 26.19% | 17.57% | 16.67% | 15.52% | 12.92% |
|  | Asian or Asian American | 2.54% | 2.38% | 1.35% | 2.85% | 1.72% | 2.81% |
|  | American Indian or Alaska Native | 3.81% | 3.57% | 4.05% | 3.66% | 3.45% | 3.93% |
|  | Native Hawaiian or Other Pacific Islander | 0.42% | 0.00% | 0.00% | 0.41% | 0.00% | 0.56% |
|  | mixed race | 8.05% | 7.14% | 4.05% | 8.94% | 5.17% | 8.99% |
|  | other / I wish not to respond | 4.66% | 7.14% | 2.70% | 6.10% | 3.45% | 5.06% |
|  | Missing | 0.42% | 1.19% | 2.70% | 0.00% | 1.72% | 0.00% |
|  | | | | | |  |  |

| Table S11: *Group Comparisons of Sample 4* | | | | | | | |
| --- | --- | --- | --- | --- | --- | --- | --- |
|  | Retained | | Removed | |  |  |  |
|  | *M* | *SE* | *M* | *SE* | *t* | *p* |  |
| Age | 20.60 | 0.30 | 20.25 | 0.36 | -0.745 | .457 |  |
| LPFS-BF 2.0 Total | 23.75 | 0.52 | 24.85 | 0.98 | 0.991 | .324 |  |
| DDSM-5-TR CC PF | 2.20 | 0.08 | 2.54 | 0.13 | 2.252 | .026 |  |
| PID5BF+M Negative Affectivity | 4.53 | 0.10 | 4.57 | 0.17 | 0.239 | .812 |  |
| PID5BF+M Detachment | 3.61 | 0.08 | 4.09 | 0.16 | 2.644 | .009 |  |
| PID5BF+M Antagonism | 3.05 | 0.07 | 3.83 | 0.16 | 4.506 | < .001 |  |
| PID5BF+M Disinhibition | 3.77 | 0.08 | 4.19 | 0.14 | 2.649 | .009 |  |
| PID5BF+M Anankastia | 4.11 | 0.11 | 4.22 | 0.15 | 0.566 | .572 |  |
| PID5BF+M Psychoticism | 3.63 | 0.10 | 4.07 | 0.16 | 2.352 | .020 |  |
| SOS-10 Total | 48.27 | 0.85 | 44.29 | 1.57 | -2.222 | .028 |  |
| WHOQOL-BREF Physical | 104.54 | 1.21 | 96.86 | 1.99 | -3.306 | .001 |  |
| WHOQOL-BREF Psychological | 76.46 | 1.19 | 76.48 | 2.03 | 0.008 | .994 |  |
| WHOQOL-BREF Social Relationships | 43.64 | 0.62 | 40.76 | 1.19 | -2.148 | .034 |  |
| WHOQOL-BREF Environment | 115.75 | 1.42 | 108.38 | 2.80 | -2.344 | .021 |  |
| IDAS-II General Depression | 50.69 | 1.04 | 51.61 | 1.68 | 0.465 | .643 |  |
| IDAS-II Dysphoria | 24.28 | 0.60 | 24.55 | 1.03 | 0.225 | .823 |  |
| IDAS-II Lassitude | 15.64 | 0.38 | 15.90 | 0.66 | 0.349 | .728 |  |
| IDAS-II Insomnia | 14.75 | 0.39 | 15.45 | 0.68 | 0.901 | .369 |  |
| IDAS-II Suicidality | 8.71 | 0.30 | 11.78 | 0.64 | 4.361 | < .001 |  |
| IDAS-II Appetite Loss | 7.06 | 0.22 | 7.62 | 0.36 | 1.335 | .184 |  |
| IDAS-II Appetite Gain | 6.72 | 0.20 | 7.35 | 0.33 | 1.647 | .102 |  |
| IDAS-II Well-Being | 21.28 | 0.43 | 21.16 | 0.77 | -0.129 | .897 |  |
| IDAS-II Ill Temper | 8.97 | 0.26 | 11.07 | 0.55 | 3.383 | < .001 |  |
| IDAS-II Mania | 10.52 | 0.31 | 11.49 | 0.57 | 1.496 | .137 |  |
| IDAS-II Euphoria | 8.43 | 0.25 | 10.49 | 0.56 | 3.383 | < .001 |  |
| IDAS-II Panic | 14.28 | 0.42 | 17.44 | 0.86 | 3.309 | .001 |  |
| IDAS-II Social Anxiety | 14.49 | 0.42 | 14.22 | 0.69 | -0.334 | .739 |  |
| IDAS-II Claustrophobia | 8.51 | 0.30 | 10.57 | 0.60 | 3.090 | .002 |  |
| IDAS-II Traumatic Intrusions | 7.80 | 0.27 | 9.01 | 0.47 | 2.251 | .026 |  |
| IDAS-II Traumatic Avoidance | 9.13 | 0.28 | 9.60 | 0.51 | 0.810 | .419 |  |
| IDAS-II Checking | 7.13 | 0.22 | 7.43 | 0.38 | 0.671 | .504 |  |
| IDAS-II Ordering | 9.80 | 0.30 | 11.21 | 0.59 | 2.145 | .034 |  |
| IDAS-II Cleaning | 12.11 | 0.37 | 15.07 | 0.79 | 3.418 | < .001 |  |
| KSE-G Exaggerating Positive Qualities | 3.57 | 0.05 | 3.01 | 0.10 | -4.847 | < .001 |  |
| KSE-G Minimizing Negative Qualities | 1.72 | 0.05 | 2.15 | 0.11 | 3.477 | < .001 |  |
| IS Total Indecisiveness | 61.69 | 0.89 | 59.80 | 2.32 | -0.761 | .449 |  |

| Table S12: *Group Comparisons of Sample 4* | | | | | | | |
| --- | --- | --- | --- | --- | --- | --- | --- |
|  | Continued | | Dropout | |  |  |  |
|  | *M* | *SE* | *M* | *SE* | *t* | *p* |  |
| Age | 20.85 | 0.64 | 20.41 | 0.25 | -0.640 | .524 |  |
| LPFS-qIAT | -0.38 | 0.06 | -0.37 | 0.03 | 0.145 | .885 |  |
| LPFS-BF 2.0 Total | 22.64 | 0.99 | 24.47 | 0.51 | 1.638 | .104 |  |
| DDSM-5-TR CC PF | 2.13 | 0.14 | 2.34 | 0.08 | 1.320 | .189 |  |
| PID5BF+M Negative Affectivity | 4.23 | 0.18 | 4.63 | 0.10 | 1.969 | .051 |  |
| PID5BF+M Detachment | 3.56 | 0.17 | 3.78 | 0.08 | 1.183 | .239 |  |
| PID5BF+M Antagonism | 3.05 | 0.13 | 3.31 | 0.13 | 1.633 | .105 |  |
| PID5BF+M Disinhibition | 3.60 | 0.14 | 3.96 | 0.08 | 2.192 | .030 |  |
| PID5BF+M Anankastia | 4.01 | 0.22 | 4.18 | 0.09 | 0.720 | .473 |  |
| PID5BF+M Psychoticism | 3.44 | 0.17 | 3.84 | 0.09 | 2.056 | .042 |  |
| SOS-10 Total | 49.73 | 1.66 | 46.47 | 0.85 | -1.748 | .083 |  |
| WHOQOL-BREF Physical | 103.73 | 2.17 | 102.16 | 1.20 | -0.632 | .529 |  |
| WHOQOL-BREF Psychological | 75.30 | 2.35 | 76.81 | 1.25 | 0.569 | .570 |  |
| WHOQOL-BREF Social Relationships | 44.05 | 1.19 | 42.54 | 0.63 | -1.128 | .261 |  |
| WHOQOL-BREF Environment | 113.30 | 2.98 | 113.97 | 1.42 | 0.203 | .839 |  |
| IDAS-II General Depression | 48.89 | 1.89 | 51.53 | 1.00 | 1.239 | .218 |  |
| IDAS-II Dysphoria | 23.11 | 1.12 | 24.72 | 0.58 | 1.278 | .204 |  |
| IDAS-II Lassitude | 14.95 | 0.75 | 15.93 | 0.36 | 1.191 | .236 |  |
| IDAS-II Insomnia | 15.07 | 0.77 | 14.89 | 0.37 | -0.211 | .833 |  |
| IDAS-II Suicidality | 8.93 | 0.57 | 9.69 | 0.33 | 1.149 | .253 |  |
| IDAS-II Appetite Loss | 7.42 | 0.45 | 7.14 | 0.21 | -0.569 | .571 |  |
| IDAS-II Appetite Gain | 6.46 | 0.37 | 7.01 | 0.19 | 1.328 | .187 |  |
| IDAS-II Well-Being | 21.85 | 0.83 | 21.06 | 0.42 | -0.854 | .395 |  |
| IDAS-II Ill Temper | 8.71 | 0.50 | 9.76 | 0.30 | 1.790 | .076 |  |
| IDAS-II Mania | 10.35 | 0.60 | 10.89 | 0.31 | 0.799 | .426 |  |
| IDAS-II Euphoria | 8.38 | 0.45 | 9.14 | 0.28 | 1.442 | .152 |  |
| IDAS-II Panic | 14.43 | 0.85 | 15.30 | 0.44 | 0.917 | .361 |  |
| IDAS-II Social Anxiety | 14.18 | 0.81 | 14.49 | 0.40 | 0.352 | .725 |  |
| IDAS-II Claustrophobia | 8.38 | 0.59 | 9.25 | 0.31 | 1.303 | .195 |  |
| IDAS-II Traumatic Intrusions | 7.85 | 0.48 | 8.20 | 0.27 | 0.633 | .528 |  |
| IDAS-II Traumatic Avoidance | 8.65 | 0.55 | 9.43 | 0.28 | 1.275 | .205 |  |
| IDAS-II Checking | 7.66 | 0.43 | 7.07 | 0.21 | -1.250 | .214 |  |
| IDAS-II Ordering | 9.80 | 0.58 | 10.28 | 0.30 | 0.734 | .464 |  |
| IDAS-II Cleaning | 11.92 | 0.65 | 13.16 | 0.40 | 1.621 | .107 |  |
| KSE-G Exaggerating Positive Qualities | 3.58 | 0.10 | 3.38 | 0.05 | -1.797 | .075 |  |
| KSE-G Minimizing Negative Qualities | 1.67 | 0.10 | 1.88 | 0.06 | 1.859 | .065 |  |
| IS Total Indecisiveness | 58.28 | 1.65 | 62.11 | 1.04 | 1.959 | .052 |  |

| Table S13: *Group Comparisons of Sample 4* | | | | | | | |
| --- | --- | --- | --- | --- | --- | --- | --- |
|  | Retained Continued | | Retained Dropout | |  |  |  |
|  | *M* | *SE* | *M* | *SE* | *t* | *p* |  |
| Age | 21.33 | 0.79 | 20.37 | 0.30 | -1.136 | .259 |  |
| LPFS-BF 2.0 Total | 22.03 | 1.01 | 24.32 | 0.59 | 1.948 | .054 |  |
| DDSM-5-TR CC PF | 1.93 | 0.14 | 2.29 | 0.09 | 2.137 | .035 |  |
| PID5BF+M Negative Affectivity | 4.26 | 0.20 | 4.61 | 0.11 | 1.558 | .122 |  |
| PID5BF+M Detachment | 3.41 | 0.17 | 3.67 | 0.09 | 1.384 | .170 |  |
| PID5BF+M Antagonism | 2.95 | 0.14 | 3.08 | 0.09 | 0.827 | .410 |  |
| PID5BF+M Disinhibition | 3.55 | 0.16 | 3.84 | 0.09 | 1.608 | .111 |  |
| PID5BF+M Anankastia | 3.97 | 0.25 | 4.16 | 0.11 | 0.705 | .483 |  |
| PID5BF+M Psychoticism | 3.32 | 0.17 | 3.73 | 0.11 | 2.030 | .045 |  |
| SOS-10 Total | 51.36 | 1.72 | 47.25 | 0.94 | -2.075 | .041 |  |
| WHOQOL-BREF Physical | 106.41 | 2.27 | 103.93 | 1.42 | -0.927 | .356 |  |
| WHOQOL-BREF Psychological | 76.69 | 2.72 | 76.38 | 1.50 | -0.099 | .921 |  |
| WHOQOL-BREF Social Relationships | 45.59 | 1.19 | 43.01 | 0.73 | -1.844 | .068 |  |
| WHOQOL-BREF Environment | 118.35 | 3.07 | 114.90 | 1.59 | -0.997 | .321 |  |
| IDAS-II General Depression | 49.09 | 2.01 | 51.19 | 1.21 | 0.894 | .374 |  |
| IDAS-II Dysphoria | 23.20 | 1.19 | 24.63 | 0.69 | 1.038 | .302 |  |
| IDAS-II Lassitude | 15.05 | 0.82 | 15.82 | 0.42 | 0.839 | .404 |  |
| IDAS-II Insomnia | 15.65 | 0.86 | 14.46 | 0.43 | -1.241 | .218 |  |
| IDAS-II Suicidality | 8.18 | 0.54 | 8.89 | 0.36 | 1.102 | .273 |  |
| IDAS-II Appetite Loss | 7.55 | 0.51 | 6.90 | 0.24 | -1.154 | .252 |  |
| IDAS-II Appetite Gain | 6.41 | 0.41 | 6.82 | 0.23 | 0.859 | .393 |  |
| IDAS-II Well-Being | 22.97 | 0.88 | 20.71 | 0.48 | -2.236 | .028 |  |
| IDAS-II Ill Temper | 8.44 | 0.53 | 9.15 | 0.34 | 1.129 | .261 |  |
| IDAS-II Mania | 10.09 | 0.63 | 10.66 | 0.36 | 0.790 | .432 |  |
| IDAS-II Euphoria | 7.90 | 0.42 | 8.61 | 0.30 | 1.382 | .170 |  |
| IDAS-II Panic | 13.75 | 0.82 | 14.45 | 0.48 | 0.729 | .468 |  |
| IDAS-II Social Anxiety | 14.72 | 0.90 | 14.41 | 0.48 | -0.306 | .760 |  |
| IDAS-II Claustrophobia | 8.00 | 0.63 | 8.68 | 0.35 | 0.949 | .345 |  |
| IDAS-II Traumatic Intrusions | 7.54 | 0.48 | 7.89 | 0.31 | 0.601 | .549 |  |
| IDAS-II Traumatic Avoidance | 8.45 | 0.58 | 9.35 | 0.32 | 1.351 | .180 |  |
| IDAS-II Checking | 7.81 | 0.45 | 6.91 | 0.25 | -1.759 | .082 |  |
| IDAS-II Ordering | 9.57 | 0.63 | 9.88 | 0.34 | 0.432 | .667 |  |
| IDAS-II Cleaning | 11.26 | 0.62 | 12.38 | 0.44 | 1.474 | .143 |  |
| KSE-G Exaggerating Positive Qualities | 3.75 | 0.10 | 3.51 | 0.06 | -2.148 | .034 |  |
| KSE-G Minimizing Negative Qualities | 1.60 | 0.09 | 1.76 | 0.06 | 1.481 | .141 |  |
| IS Total Indecisiveness | 59.78 | 1.60 | 62.32 | 1.06 | 1.325 | .188 |  |

| Table S14: *Group Comparisons of Sample 4 Informant Ratings* | | | | | | | |
| --- | --- | --- | --- | --- | --- | --- | --- |
|  | Retained | | Removed | |  |  |  |
|  | *M* | *SE* | *M* | *SE* | *t* | *p* |  |
| LPFS-BF 2.0 Total | 21.96 | 0.47 | 21.83 | 1.23 | -0.095 | .925 |  |
| PID5BF+M Negative Affectivity | 4.52 | 0.10 | 4.22 | 0.22 | -1.227 | .225 |  |
| PID5BF+M Detachment | 3.37 | 0.07 | 3.51 | 0.19 | 0.682 | .498 |  |
| PID5BF+M Antagonism | 2.87 | 0.07 | 2.92 | 0.23 | 0.208 | .836 |  |
| PID5BF+M Disinhibition | 3.54 | 0.08 | 3.24 | 0.19 | -1.460 | .150 |  |
| PID5BF+M Anankastia | 4.22 | 0.10 | 3.98 | 0.25 | -0.880 | .383 |  |
| PID5BF+M Psychoticism | 3.38 | 0.09 | 3.00 | 0.20 | -1.753 | .085 |  |

| Table S15  *Descriptive Statistics of All Samples without Dropping Fast Responders* | | | | | | | |
| --- | --- | --- | --- | --- | --- | --- | --- |
|  | | Sample 1 | Sample 2 | Sample 3 (Time 1) | Sample 3 (Time 2) | Sample 4 (Time 1) | Sample 4 (Time 2) |
|  |  | (*N* = 1033) | (*N* = 790) | (*N* = 287) | (*N* = 161) | (*N* = 320) | (*N* = 74) |
| Age, μ (SD) | | 20.97 (4.52) | 21.25 (5.01) | 21.38 (6.09) | 21.31 (6.77) | 20.49 (4.26) | 20.88 (5.43) |
| Sex (%) | |  |  |  |  |  |  |
|  | Female | 83.09% | 82.41% | 86.16% | 85.71% | 85.09% | 87.67% |
|  | Male | 16.43% | 17.47% | 13.50% | 13.04% | 14.29% | 12.33% |
|  | Other / I wish not to respond | 0.48% | 0.13% | 0.35% | 1.24% | 0.62% | 0.00% |
| Ethnicity (%) | |  |  |  |  |  |  |
|  | Hispanic / Latine | 31.69% | 35.28% | 28.67% | 34.38% | 33.23% | 41.10% |
|  | Non-Hispanic / Latine | 68.31% | 64.72% | 71.33% | 65.63% | 66.77% | 58.90% |
| Race (%) | |  |  |  |  |  |  |
|  | White or Caucasian | 65.33% | 68.15% | 63.29% | 67.72% | 62.93% | 69.86% |
|  | Black or African American | 17.77% | 15.16% | 21.33% | 17.72% | 17.45% | 19.18% |
|  | Asian or Asian American | 2.83% | 2.29% | 4.55% | 3.17% | 2.49% | 1.37% |
|  | American Indian or Alaska Native | 2.25% | 2.93% | 3.15% | 3.17% | 3.74% | 2.74% |
|  | Native Hawaiian or Other Pacific Islander | 0.29% | 0.89% | 0.35% | 0.00% | 0.31% | 0.00% |
|  | mixed race | 7.32% | 5.10% | 4.20% | 3.80% | 7.79% | 2.74% |
|  | other / I wish not to respond | 4.20% | 5.48% | 3.15% | 4.43% | 5.30% | 4.11% |
|  | | | | | | | |

| Table S16.  *Correlation Coefficients Depicting the Convergent Validity of the LPFS-qIAT with Other Measures of Personality Functioning Impairment and the LPFS-qIAT’s Criterion-Related Validity without Dropping Fast Responders* | | | | | |
| --- | --- | --- | --- | --- | --- |
| Measure | Method | Sample 1 | Sample 2 | Sample 3 | Sample 4 |
|  |  | (*N* = 1033) | (*N* = 790) | (*N* = 287) | (*N* = 320) |
| LPFS-BF 2.0 Total | Self-Report | **.137** (.072, .201) | -- | -- | .080 (-.059, .215) |
|  | Informant^†^ | -- | -- | .022 (-.053, .101) | |
| LPFS-SR Total | Self-Report | **.157** (.095, .218) | **.135** (.007, .143) | .103 (-.012, .213) | -- |
| DSM-5-TR CC PF | Self-Report | -- | -- | -- | .093 (-.027, .214) |
| Negative Affectivity | Self-Report | **.065** (.006, .123) | -.005 (-.070, .059) | .006 (-.103, .105) | .042 (-.073, .164) |
| Detachment | Self-Report | **.111** (.051, .167) | **.138** (.066, .208) | -.026 (-.134, .086) | .072 (-.045, .189) |
| Antagonism | Self-Report | **.093** (.034, .151) | .054 (-.013, .122) | .005 (-.108, .121) | **.128** (.011, .247) |
| Disinhibition | Self-Report | **.103** (.043, .162) | .069 (-.001, .136) | -.044 (-.158, .057) | .089 (-.023, .202) |
| Anankastia | Self-Report | **.059** (.001, .117) | .046 (-.024, .113) | .073 (-.045, .191) | -.060 (-.167, .058) |
| Psychoticism | Self-Report | **.062** (.002, .121) | .062 (-.006, .130) | .039 (-.061, .137) | .031 (-.076, .146) |
| SOS-10 | Self-Report | -- | -- | -- | -.118 (-.234, .007) |
| WHOQOL-BREF Physical | Self-Report | -- | -- | -- | -.089 (-.200, .023) |
| WHOQOL-BREF Psychological | Self-Report | -- | -- | -- | -.001 (-.108, .104) |
| WHOQOL-BREF Social Relationships | Self-Report | -- | -- | -- | -.023 (-.138, .093) |
| WHOQOL-BREF Environment | Self-Report | -- | -- | -- | -.089 (-.203, .025) |
| *Note*. Bold indicates statistical significance based on 95% confidence intervals from 5000 bootstrap replicates. Sample 1 personality traits scores were obtained using the PID5BF+ (Kerber et al., 2019) whereas trait scores for all other samples obtained using the PID5BF+M (Bach et al., 2020). ^†^627 informant ratings of 380 participants. | | | | | |

| Table S17.  *Correlation Coefficients Depicting the Discriminant Validity of the LPFS qIAT without Dropping Fast Responders* | | | | |
| --- | --- | --- | --- | --- |
| Discriminant Measure | Sample 1 | Sample 2 | Sample 3 | Sample 4 |
|  | (*N* = 1033) | (*N* = 790) | (*N* = 287) | (*N* = 320) |
| Age | **-.073** (-.132, -.014) | **-.075** (-.143, -.007) | -.047 (-.133, .044) | **-.079** (-.152, -.002) |
| Extraversion IAT | -- | -.032 (-.101, .038) | -- | -- |
| IDAS-II |  |  |  |  |
| General Depression | -- | -- | -- | .031 (-.081, .143) |
| Dysphoria | -- | -- | -- | .022 (-.089, .135) |
| Lassitude | -- | -- | -- | .062 (-.051, .173) |
| Insomnia | -- | -- | -- | .038 (-.067, .145) |
| Suicidality | -- | -- | -- | .051 (-.067, .174) |
| Appetite Loss | -- | -- | -- | .004 (-.108, .120) |
| Appetite Gain | -- | -- | -- | -.005 (-.107, .097) |
| Well-Being | -- | -- | -- | -.030 (-.140, .075) |
| Ill Temper | -- | -- | -- | .090 (-.032, .218) |
| Mania | -- | -- | -- | -.019 (-.131, .100) |
| Euphoria | -- | -- | -- | .051 (-.058, .163) |
| Panic | -- | -- | -- | .061 (-.063, .185) |
| Social Anxiety | -- | -- | -- | .004 (-.098, .109) |
| Claustrophobia | -- | -- | -- | .038 (-.076, .162) |
| Traumatic Intrusions | -- | -- | -- | .012 (-.108, .134) |
| Traumatic Avoidance | -- | -- | -- | -.002 (-.118, .119) |
| Checking | -- | -- | -- | -.023 (-.131, .092) |
| Ordering | -- | -- | -- | .018 (-.092, .135) |
| Cleaning | -- | -- | -- | .032 (-.081, .149) |
| KSE-G |  |  |  |  |
| Exaggerating Positive Qualities | -- | -- | -- | -.097 (-.209, .022) |
| Minimizing Negative Qualities | -- | -- | -- | **.138** (.012, .268) |
| Indecisiveness Scale |  |  |  |  |
| Total Indecisiveness | -- | -- | -- | -.018 (-.179, .141) |
| Minimum Detectable Effect Size | .087 | .088 | .164 | .156 |
| *Note*. Bold indicates statistical significance based on 95% confidence intervals from 5000 bootstrap replicates. Minimum detectable effect sizes derived from sensitivity analysis based on sample size and two-tailed significance test given α = .05 and power of .80. | | | | |
